# A descriptive epidemiological study of the prevalence of self-reported sensory difficulties by age group, sex, education, disability, and migration status in Sweden in 2020

**DOI:** 10.1186/s12889-024-20217-1

**Published:** 2024-10-10

**Authors:** Andreea-Corina Badache, Elina Mäki-Torkko, Stephen Widen, Stefan Fors

**Affiliations:** 1 School of Health Sciences, Örebro University, Fakultetsgatan 1, Örebro, 701 82 Sweden; 2 Swedish Institute for Disability Research, Örebro, Sweden; 3 School of Medical Sciences, Örebro University, Örebro, Sweden; 4 Audiological Research Center, Faculty of Medicine, and Health, Örebro University, Örebro, Sweden; 5 Aging Research Center, Karolinska Institutet and Stockholm University, Stockholm, Sweden; 6 Centre for Epidemiology and Community Medicine, Region Stockholm, Stockholm, Sweden; 7 Department of Public Health Sciences, Stockholm University, Stockholm, Sweden

**Keywords:** Older adults, Sweden, Hearing difficulties, Prevalence, Cross-sectional

## Abstract

**Background:**

The objective of this study is to estimate the prevalence of self-reported hearing difficulties, vision difficulties and combined vision and hearing difficulties in a Swedish adult population that varies according to migration status, sex, age, disability measured by ADL and IADL and educational attainment level.

**Methods:**

The study utilised data from the Survey of Health, Ageing and Retirement in Europe, which consisted of 2257 individuals aged 60 and above that were interviewed in Sweden in the 2019/2020, SHARE wave 8. To determine the prevalence of sensory difficulties (hearing, vision and dual-sensory difficulties) among various subgroups of the population, a multinomial logistic regression was used. The results of these analyses are presented in terms of predicted probabilities.

**Results:**

The study findings indicate that foreign-born older adults experience a lower prevalence of visual difficulties (6.2% [3.3–11.5] in comparison to their Swedish-born counterparts. Moreover, older adults with higher levels of education tend to report a lower prevalence of sensory difficulties overall. Furthermore, sex differences are apparent, with males reporting a higher prevalence of hearing difficulties (18.9% [15.5–22.8] vs. 12.8% [10.7–15.3]) and females reporting a higher prevalence of vision difficulties (12.7% [10.7–15.1] vs. 8.5%[6.8–10.5]).

**Conclusion:**

The findings highlight disparities in the prevalence and type of perceived sensory difficulties experienced by older adults, by factors such as age, sex, education and migration status. It is important to consider these demographic factors in healthcare planning and interventions aimed at mitigating sensory difficulties in the older population.

## Introduction

Ageing is associated with a slow and gradual decline in sensory functions, a critical component of older people’s health [1] and quality of life [2, 3]. Hearing and vision losses impose a significant burden for older people and may significantly affect public health as they have numerous negative effects on individuals, such as difficulty with communication [4], depression [5], poor quality of life [6], increased risk for dementia [7] and even increased mortality risk [8, 9]. Importantly, deterioration of the sensory functions are directly linked to an older person’s ability to carry out activities of daily living [10].

While on a global scale life expectancy has increased, the burden of sensory losses, such as hearing and vision loss, has also increased. Globally, according to the estimates of the World Health Organization, there are at least 2.2 billion people living with vision loss and 1.5 billion people with hearing loss [11, 12], most of whom are older people. These numbers are projected to raise over the coming years [11, 12] due to increased longevity. Hearing loss has become the third leading cause of years lived with disability, and hearing and vision losses were the largest contributors to disability in older age groups from 1990 to 2016 [13]. Vision loss is the sixth leading cause of disability worldwide [14]. Furthermore, dual-sensory loss, which refers to the presence of combined vision and hearing loss in an individual, is a growing concern in older adults [15]. However, the limited access to prevalence estimates on dual-sensory loss makes it difficult to fully understand the burden related to it, as well as the needs of this population. In the US, between 1999 and 2006, an estimated 1.5 million adults had dual-sensory loss, measured by pure-tone audiometry and visual acuity, with a prevalence of 11% in those aged 80 or older [16]. For comparison, in Canada, in a population including both middle-aged and older adults aged 45–85 years, the dual-sensory loss prevalence estimated by measuring hearing and vision (with a digital screening audiometer and binocular visual acuity using habitual correction) was estimated to be 4.1% [17].

There are geographic and sex variations in the prevalence of vision [14], hearing [18], and dual-sensory losses [19]. In both Sweden and Denmark, a previous study showed that the prevalence of hearing and vision difficulties is decreasing over time [20]. In both low and high income countries, socio-economic deprivation may be associated with higher degree of both vision [21, 22] and hearing losses [23, 24]. Regarding hearing, the association could be mediated by living and working conditions in noisy environments [25]. Furthermore, regarding both vision and hearing losses, the association with socio-economic deprivation could be through non-communicable diseases and lifestyle factors [26], such as smoking behaviours, which are associated with sensory losses. In high income countries, regardless of age, the prevalence of vision loss is higher among women compared to men [27]. Although the mechanisms that produce these sex inequalities are not well understood, these can, for example, be related to the exposure to risk factors, lifestyle behaviours and barriers hindering access to health care services [28]. In contrast, men report higher rates of hearing loss and have worse hearing pure-tone thresholds than women [29]. This difference is believed to be influenced by factors including hormones such as oestrogen [30]. Moreover, men are less likely to talk about their hearing difficulties and seek help, which could be due to gender stereotypes [31]. Men are moreover more likely to be exposed to noise-induced hearing loss due to occupational and recreational activities [30].

Previous studies have shown that there are differences in the prevalence of hearing [32] and vision [33] losses based on ethnicity. Additionally, several studies have demonstrated that the prevalence of these conditions can also vary according to socio-economic factors such as educational level [34]. According to Statistics Sweden, 20% of the total Swedish population is foreign-born [35]. Among those aged 65 and older, there are educational differences: 36% of foreign-born individuals in this age group have achieved a high level of education, defined as completing at least 3 years after high school. This compares to 41% of Swedes in the same age group who have attained a similar level of education [36]. Over the years, immigration to Sweden has fluctuated, with significant influxes of labour immigrants in the 1960s, Iranian refugees in the 1980s, and individuals from former Yugoslavia in the 1990s. Among labour immigrants there were more men than women [37].

Although it is well documented that health disparities exist based on migration status and socioeconomic status, and that prevalence estimates are important for understanding the distribution and impact of a particular health condition within a population, there is still a lack of prevalence estimates, especially for specific sub-groups based on age, sex, migration status, educational attainment and disability level, for example. The lack of prevalence estimates in various sub-groups for sensory losses can impede efforts to design and target public health interventions and monitor their impact on health disparities. To our knowledge, there is no recent study in Sweden that has examined the relationship between sensory difficulty in older people, that is individuals aged 60 years or older [38], and migration status, sex, disability, and educational attainment, and whether any associated disparities exist. Therefore, the aim of this study is to estimate the prevalence of self-reported hearing difficulties, vision difficulties and combined visual and hearing difficulties in a Swedish older population, which has different migration status, sex, age, disability level and educational attainment level. Based on the previous literature, we hypothesize that there are significant disparities in the prevalence of self-reported hearing difficulties, vision difficulties, and combined visual and hearing difficulties among the Swedish adult population, and that these disparities are associated with migration status, sex, disability, and educational attainment.

## Methods

### Data

The Survey of Health, Ageing and Retirement in Europe [39] is a large-scale, cross-national, and longitudinal study that aims to investigate the life-course effects of health, social, economic, and environmental policies on European citizens aged 50 years and older and is based on representative samples drawn from a representative population; the sampling designs vary across countries and are based on random or stratified sampling. However, individuals living in institutions are not part of the target population. Yet, individuals from the longitudinal cohort who transition into institutions will continue to be followed [39]. In Sweden, the sampling design for the SHARE survey uses a multi-stage stratified approach, which involves dividing the country into several strata to ensure the representativeness of different geographical areas. From Wave 5 onwards, the central population register has provided a direct sampling frame that makes the multistage sampling design unnecessary as individuals can be drawn directly from the central population register [40].

In 2022, SHARE publicly released Wave 8 data collected in 2019/2020; however, there were several previous waves of data collection, with the first wave taking place in 2004 and subsequent waves occurring every two years. Most interviews take place in the participants’ homes, but when respondents are unable to participate due to health reasons, proxy interviews are conducted with relatives or caregivers. In Wave 8 across most countries, there was a suspension of face-to-face interviews in March 2020 due to the COVID-19 pandemic. The impact of this suspension on retention rates varied by country, depending on how far along fieldwork was at the time of the suspension. In response to the pandemic, a special SHARE Corona survey (SCS) was conducted by telephone in summer 2020, which helped to stabilise retention rates in most countries and even surpass pre-pandemic levels. Moreover, to maintain representations of the younger age cohorts and compensate for attrition, regular refreshment samples were also drawn. In wave 8, Sweden, among 15 other countries, recruited refreshment samples. The response rate for SHARE Wave 8 is not yet available; however, for Sweden, for the refreshment sample, the household response rate was 17.3% out of 875 households that were estimated to be eligible. When it comes to the longitudinal sample (the participants that completed at least 1 interview in the previous waves), the response rate was 61.5%.

Since the aim of this study is to analyse a specific point in time, the data are analysed as a cross-sectional design with data from Wave 8, which were gathered in 2019 and 2020. The language of the interview was Swedish for all the participants.

We chose SHARE for our study as it is based on random samples of the older population in Sweden, which allows us to make statistical inferences to our target population.

### Measures

#### Sensory functions

*Perceived* hearing and vision difficulties were assessed through self-reported measures. Specifically, the participants were asked to rate their hearing as well as distance and reading vision (using glasses/contact lenses or a hearing aid as usual) on a 5-point scale (1 = excellent, 2 = very good, 3 = good, 4 = fair, 5 = poor). For vision, a new variable was created that combined ratings from distance and reading vision. Based on the results from the hearing and vision variables, a new outcome variable with four categories was created to categorise the participants according to their self-reported hearing and vision abilities. Participants who self-reported fair or poor hearing and vision were categorised as having dual-sensory difficulties. Those who reported only fair or poor hearing were categorised as having hearing difficulties, and those who reported only fair or poor vision were categorised as having vision difficulties. Finally, participants who reported good, very good or excellent hearing and vision were categorised as having no sensory difficulties.

*Migration status* based on the participants place of birth was dichotomised according to participants’ self-reports regarding being Swedish born or foreign-born.

*Disability* is defined in this study as the difficulty in performing activities of daily living (ADL) and instrumental activities of daily living (IADL). ADL are basic self-care activities, such as dressing, eating, and bathing, that are necessary for independent living. IADL are more complex tasks that involve managing finances, shopping, and taking medication, among others. The participants in this study reported their ability to perform ADL and IADL tasks on a self-reported scale, adapted from Katz et al. (1970) [41] and Lawton and Brody (1969) [42], respectively. Those who reported difficulty in performing one or more tasks without help were classified as having ADL and/or IADL limitations. Both ADL and IADL were divided into two categories: those with no limitations and those with limitations in one or more tasks.

*Education* was assessed through self-report and classified into three categories based on the International Standard Classification of Education (ISCED) 1997 levels [43]: primary and lower secondary (ISCED 0–2), upper secondary (ISCED 3–4), and tertiary (ISCED 5–6).

#### Age and sex

Age was divided into two categories, 60 to 69 years old and 70 years and older, to capture the higher prevalence of sensory difficulties in the older age groups. According to previous research, hearing [44] and vision [45] losses are more common in older adults, particularly those aged 70 and above. Sex was categorised as female or male. Participants under the age of 60 were excluded and the analysis was conducted for complete cases only: thus, participants with missing data were excluded from the analysis. The decision to exclude individuals aged 60 and under from our study is to focus specifically on old-age sensory difficulties in alignment with the United Nations’ definition of older adults [38]. Our primary aim was to investigate sensory functions in this specific older population. Age and sex were included as covariates in all the models.

### Data analysis

The data were analysed using STATA 17.0, and the prevalence of self-reported sensory difficulties by age, migration status, sex, and education were estimated. This was done by calculating the prevalence of individuals in each subgroup who report having each type of sensory difficulty. Multinomial logistic regression analysis was used to estimate the prevalence of sensory difficulties that are categorized into four distinct groups as described above for different subgroups of the population. This method is particularly appropriate for our analysis as it can well handle the nominal nature of our outcome variable, which lacks an inherent order among the categories. Multinomial logistic regression is suitable for modelling the probabilities of belonging to each sensory category and identifying significant predictors, while adjusting for covariates. By utilizing this approach, we aim to gain a deeper understanding of how different factors are associated with the probability of belonging to each of the four sensory categories. The results of these analyses are presented in terms of predicted probabilities, which are calculated using marginal standardisation [46]. This method allows the researchers to obtain estimates of the probability of an outcome (e.g., having a particular type of sensory difficulty) for each level of the predictor variables (e.g., age, sex, education) while controlling for the distribution of the other variables in the sample. By calculating predicted probabilities, the results can be made more intuitively interpretable. Moreover, the predicted probabilities allow us to assess the adjusted prevalence of the outcomes in different sub-groups of the population. To estimate the prevalence of sensory difficulties, the use of predicted probabilities multiplied by 100 was calculated, to report results as percentages. In our analysis, age and sex are included as covariates in all models as it is well-documented that the prevalence of sensory functions varies across sex and age groups. Thus, adjusting for age and sex eliminates the likelihood that observed group differences could be attributed to differences in the sex and age composition between the groups.

To estimate differences in prevalence between different subgroups of the population, we also calculated the average marginal effect (AME) as an absolute indicator for differences across the subgroups. AME represents the average difference in the predicted probability of each category of the dependent variable between groups defined by different values on the independent variable. While the predicted probabilities provide a summary of the predicted probability of each category of the dependent variable, the AME estimates the magnitude of the differences between the predicted probabilities of different groups.

## Results

### Descriptive statistics

A total of 2257 individuals with an age range of 60 to 100 were interviewed in Sweden in the 2019/2020 SHARE wave 8. Among them there was a slightly higher representation of female participants compared to male participants (50.7% vs. 49.3%), with a greater proportion of Swedish-born individuals compared to foreign-born individuals (91.4% vs. 8.6%). The distribution across different levels of education is balanced. Primary and lower secondary levels account for 32.8% of the educational background, closely followed by upper secondary education at 34.3%. Tertiary education is represented by 32.9%.

The weighted prevalence of people reporting vision, hearing, and dual-sensory difficulties was lower in groups with higher education, and vice versa (Table 1). Females had a slightly higher prevalence than men in reported dual-sensory difficulties (5.9% vs. 6%) and visual difficulties (12.7% vs. 8.5%) while men reported a higher prevalence of hearing difficulties (18.9% vs. 12.8%) compared to women. Foreign-born adults reported a higher prevalence of hearing difficulties (23.4% vs. 15.1%); however, they reported a lower prevalence of vision difficulties (6.2% vs. 10.9%) and dual-sensory difficulties (4.6% vs. 6.1%) compared to Swedish-born older adults. Table 1 presents the weighted prevalence of sensory difficulties stratified by age, sex, migration status, education, and ADL and IADL.

**Table 1:** Descriptive statistics for older adults aged 60 and above in Sweden

Sample characteristics	Vision difficulties [95% CI]	Hearing difficulties [95% CI]	Dual-Sensory difficulties [95% CI]	No sensory difficulty [95% CI]	Sample proportion
**Age (%)**					
60 to 69	7.3% [5.3–10]	14% [10.4–18.5]	3% [1.7–5.1]	75.7% [70.7–80.1]	45.6%
70 and above	**13.3% [11.7–15.3]**	17.4% [15.5–19.4]	**8.4% [7.1–10]**	**60.8% [58.3–63.3]**	54.4%
Total (*N* = 2257)	10.6%	15.8%	5.9%	67.6%	
**Sex (%)**					
Female	**12.7%** [10.7–15.1]	**12.8%** [10.7–15.3]	6% [4.6–7.9]	68.4% [65.1–71.6]	50.7%
Male	8.5% [6.8–10.5]	18.9% [15.5–22.8]	5.9% [4.5–7.5]	66.8% [62.4–70.9]	49.3%
Total (*N* = 2257)	10.6%	15.8%	5.9%	67.6%	
**Migration status (%)**					
Swedish born	10.9% [9.4–12.5]	15.1% [13.3–17.2]	6.1% [5.0-7.4]	67.9% [65.2–70.5]	91,4%
Foreign born	6.2% [3.3–11.5]	23.4% [12.7–38.9]	4.6% [2.3-9]	65.7% [51.2–77.4]	8.6%
Total (*N* = 2222)	10.5%	15.8%	5.9%	67.7%	
**Education (%)**					
Primary and lower secondary	15.7% [13.0-18.8]	20% [16.9–23.5]	10.5% [8.0-13.8]	53.8% [49.6–57.9]	32.8%
Upper secondary	**11.5% [9.1–14.4]**	15.8% [13.0–19.0]	**5.7% [4.1-8]**	**67% [63-70.8]**	34.3%
Tertiary	**9.6% [7.4–12.2]**	**13.9% [11.4–16.9]**	**5.5% [3.9–7.7]**	**71.0% [67.2–74.5]**	32.9%
Total (*N* = 2030)	12.2%	16.6%	7.2%	64%	
**ADL impairments**					
No	9.6% [8.2–11.2]	14.8% [12.9–16.9]	5% [4.0-6.2]	70.7% [68-73.2]	89.8%
Yes	**19.7% [14.4–26.4]**	**25.3% [16.4–36.7]**	**14.5% [10.0-20.5]**	**40.6% [33.0-48.8]**	10.2%
Total (*N* = 2256)	10.6%	15.8%	5.9%	67.6%	
**IADL impairments**					
No	9.2% [7.8–10.9]	15% [12.8–17.6]	3.8% [3-4.8]	72% [69-74.7]	85%
Yes	**18.6% [13.6–23.5]**	**20.4% [16.4–25.1]**	**18% [13.6–23.4]**	**43% [37.5–48.8]**	15%
Total (*N* = 2256)	10.6%	15.8%	5.9%	67.6%	

The significance tests were calculated with logistic regression. All statistically significant unadjusted results are marked in bold

### Sensory difficulties and sex, age, educational attainment, migration status and disability

**Fig. 1:**
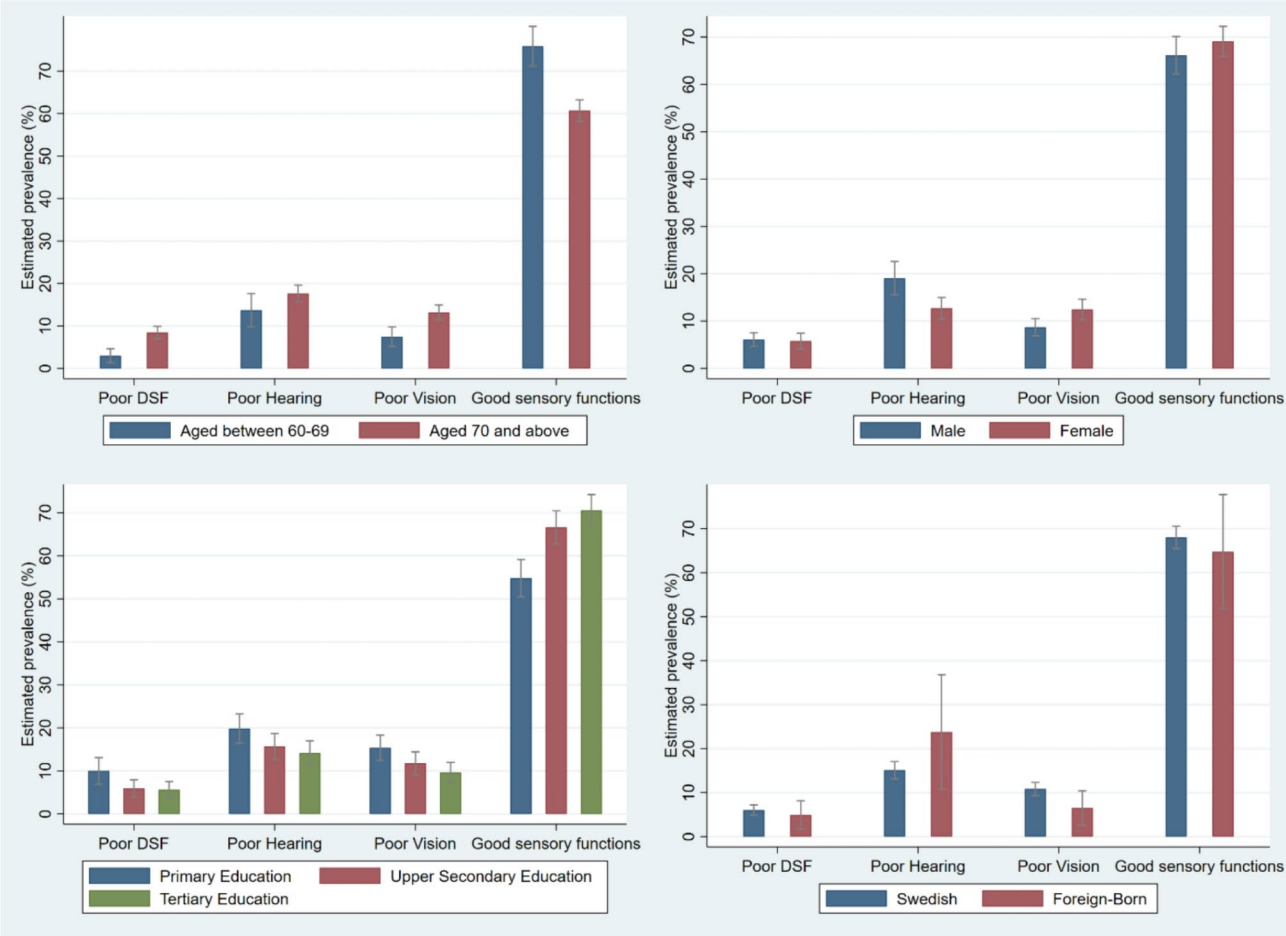
The prevalence of sensory difficulties by (**a**) age (upper left quadrant); (**b**) sex (upper right quadrant); (**c**) educational attainment (lower left quadrant); and (**d**) migration status (lower right quadrant). DSF = dual-sensory function

The prevalence of sensory difficulties by migration status, age group, sex, and education during 2019/2020 are presented in Fig. 1 with adjustments for sex and age. Swedish-born older individuals had a statistically non significant higher prevalence of perceived dual-sensory 6.1%, [CI: 5-7.4] and vision difficulties 10.9%, [CI: 9.4–12.5] compared to foreign-born individuals 4.6%, [CI: 2.3-9.] and 6.2%, [CI: 3.3–11.5], respectively). However, the prevalence of perceived poor hearing was higher in foreign-born individuals 23.4%, [CI: 12.7–38.9]) compared to Swedish-born individuals (15.1%, [CI: 13.3–17.2]), although not statistically significant. Foreign-born individuals had a statistically significant lower prevalence (4.3% points) of vision difficulties compared to Swedish-born individuals as seen in Table 2.

The prevalence of reporting sensory difficulties is higher in the older age group. Dual-sensory difficulties, poor hearing and poor vision are more likely reported by those aged 70 and above (see Fig. 1a). Compared to younger adults, those above the age of 70 have a higher prevalence of hearing difficulties 3.9% points [CI: -0.4-8.02]), vision difficulties 5.7% points [CI: 2.8–8.6]) and dual-sensory difficulties 5.5% points [CI: 3.3–7.8]. Female adults report a statistically significant lower prevalence of poor hearing – 6.4% points [CI: − 10.5 to -2,3] than male adults, but they are more likely to have a higher prevalence of vision difficulties 3.8% [CI: 0.9 to 6.6] points than men (Table 2).

Older adults with higher levels of education have a lower prevalence of sensory difficulties, as shown in Fig. 1c. For instance, those with upper secondary education have a lower prevalence of hearing 4.1% points [CI: -8.7 to 0.4], dual-sensory difficulties 4.1% points [CI: -7.8 to -0.3], and vision difficulties 3.6% points [CI: -7.5 to 0.4]. Those with tertiary education have an even lower prevalence of any sensory difficulties. Specifically, they report a lower prevalence of dual sensory difficulties by 4.4% points [CI: -8.0 to -0.7], hearing difficulties by 5.7% points [CI: -10.1 to -1.3] and vision difficulties by 5.7% points [CI: -9.5 to -2.0]. Overall, they have a higher prevalence of those reporting no sensory difficulties at all 15.8% points [CI: 10.1 to 21.4] compared to adults with upper secondary and primary education (Table 2).

**Fig. 2:**
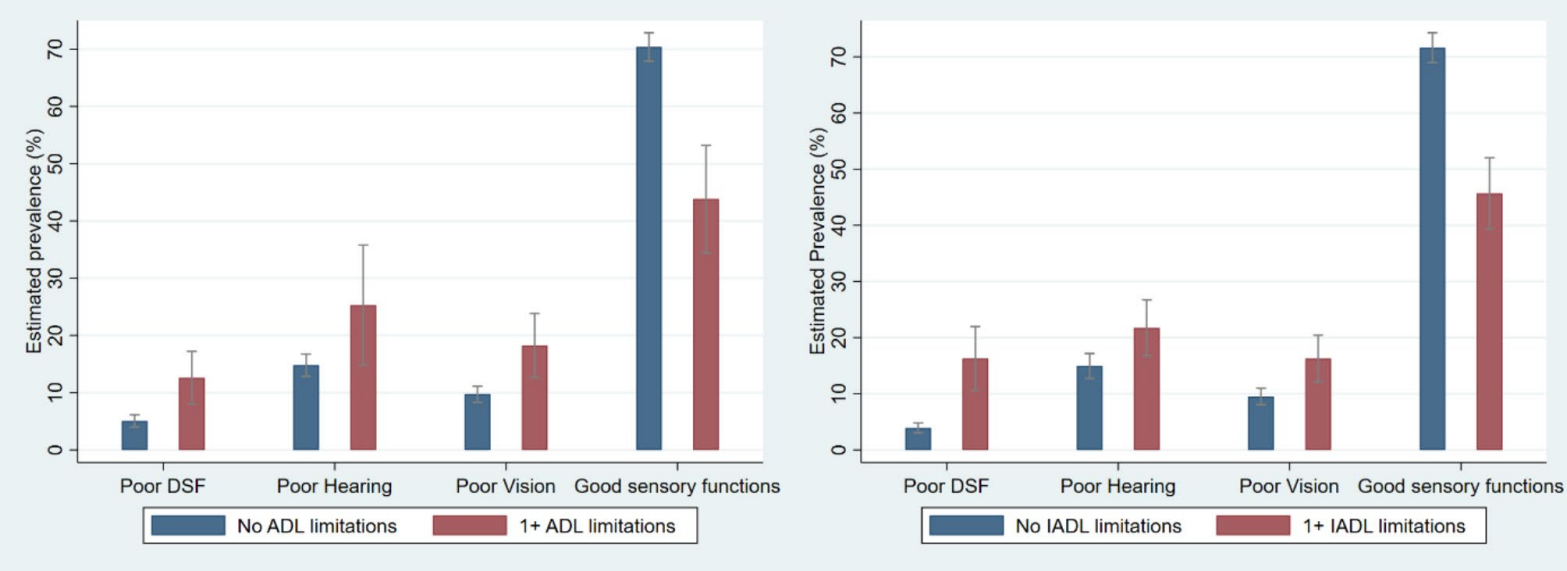
**a**) Left quadrant: The prevalence of sensory difficulties by ADL limitations; **b**) Right quadrant: the prevalence of sensory difficulties by IADL limitations. DSF = dual-sensory function

The prevalence of all the sensory difficulties is higher in those who have a higher prevalence of ADL and IADL limitations as demonstrated in Fig. 2. The highest prevalence of ADL and IADL limitations are among those who also reported a higher prevalence of hearing and vision difficulties. Specifically, 25.3% [CI: 16.4–36.7] of those with hearing difficulties and 19.7% [CI: 14.4–26.4] of those with vision difficulties report limitations in ADL, while 20.4% [CI: 16.4–25.1] of those with hearing difficulties and 18.6% [CI: 13.6–23.5] of those with vision difficulties report limitations in IADL. People who report dual-sensory difficulties have a higher prevalence of ADL 14.5% [CI: 10–20.5] and IADL 18% [CI: 13.6–23.4] limitations. The highest prevalence of ADL 25.3% [CI: 16.4–36.7] and IADL limitations 20.4% [CI: 16.8–26.7] is found in individuals who experience hearing difficulties. In Table 2, it is shown that individuals with a lower prevalence of ADL and IADL limitations also report sensory difficulties.

**Table 2:** Average marginal effects of sensory difficulties, disability level, migration status, sex, and age in older adults: results from multinominal regression analysis

Sample characteristics	Dual-Sensory difficulties	Hearing difficulties	Vision difficulties	Good hearing and vision	Total (*n*)
**Sex**					2257
Male	Reference				
Female	-0.003	-0.064**	0.038*	0.029	
**Age**					2257
60–69	Reference				
70 and above	0.055***	0.039	0.057*	-0.152***	
**Education**					2030
Low	Reference				
Medium	-0.041*	-0.041	-0.036	0.118***	
High	-0.044*	-0.057**	-0.057*	0.158***	
**ADL**					2257
No ADL	Reference				
Yes ADL	0.075***	0.105	0.085**	-0.266***	
**IADL**					2257
No IADL	Reference				
Yes IADL	0.123***	0.068**	0.068**	-0.259***	
**Migration status**					2222
Native	Reference				
Foreign-born	-0.011	0.086	-0.043*	-0.033	

All statistically significant results are in bold *p ≤ 0.05; **p ≤ 0.01; ***p ≤ 0.001

All models are adjusted for sex and age

## Discussion

This study aimed to assess the prevalence of self-reported hearing, vision, and dual-sensory difficulties among older adults in Sweden during 2019/2020 and to explore how sensory difficulties are distributed between different groups. The results showed that foreign-born older adults have a lower prevalence of visual difficulties in comparison to Swedish-born older adults. Additionally, older adults with higher levels of education have a lower reported prevalence of sensory difficulties overall. Furthermore, the prevalence of sensory difficulties tends to increase with age, and males tend to report a higher prevalence of hearing difficulties, while females report a higher prevalence of vision difficulties.

It should be noted that there are some limitations to consider when utilising SHARE data. The primary concern is the low response rate; however, in comparison to other European and US survey studies, the overall response rate of SHARE is relatively high [39]. A low response rate can potentially generate sample selection bias and limit the representativeness of the database and generalisability of results. To address these issues, SHARE employs ex-post calibrated weights, which were also used in all the analyses of this study. Due to the COVID-19 outbreak and the suspension of the fieldwork in March 2020, concerns were raised regarding the representativeness of the panel sample and the quality of data collected in Wave 8; therefore, SHARE conducted a study to investigate selective participation in Wave 8 [47]. The study found that many relevant characteristics, such as socio-demographics, were not significantly correlated with participation in wave 8. Additionally, the study found that very old adults and those living alone had a significantly higher probability to participate in Wave 8 as they frequently live alone due to a partner’s death, and are more accessible unless they have significant physical or cognitive impairments [47]. Overall, there was little evidence to suggest that health-related outcomes were significantly affected by selection bias [47]. To ensure that the sample remains representative over time, refreshment samples are continuously added in SHARE. In the wave 8, Sweden had amongst the highest retention rate from previous waves, which was close to 90% and included earlier refreshment samples. However, the response rates for the latest refreshment samples were low, meaning that there are uncertainties surrounding how well the prevalence estimates accurately represent the true prevalence in the population. If the individuals who are not responding to the survey were more (or less) likely to have sensory difficulties than those who respond, it could result in an underestimation of the prevalence [47]. Therefore, our estimates may be underestimations of the true prevalence, especially in the oldest age groups. Additionally, it is important to acknowledge the limitations associated with the subjective nature of self-assessment questions in health surveys. Individual perceptions of sensory functions can vary significantly, even when objective capabilities are similar [48]. This is particularly pertinent when comparing self-assessments with performance-based tests, which are both common methods used in surveys of older adults. Additionally, the use of self-reported data, includes the potential for recall bias, where participants may inaccurately remember or report past experiences [49]. Social desirability bias is another consideration, as participants may alter responses to align with social expectations or due to misunderstandings of the questions [49]. Such alterations can skew the results, leading to a misrepresentation of the actual sensory status. Health literacy, which encompasses an individual’s ability to obtain, process, and understand basic health information, can influence reporting accuracy as the average levels of health literacy is likely to vary across groups, this could also affect our findings, especially considering that the interviews were conducted in Swedish and people who are not fluent in Swedish were not included in the study. Future research may benefit from incorporating methods to mitigate these biases, such as using objective measures to measure sensory functions or employing strategies to enhance the clarity and understanding of survey questions. Finally, our research is based on data collected from the Swedish population, which has specific demographic, cultural, and socioeconomic characteristics. As a result, the findings of our study may not be directly applicable to other populations in different geographical areas or cultural contexts. The conclusions drawn from our study are primarily relevant to the Swedish context and may not necessarily reflect the experiences or trends in other regions or cultures.

The results of this study are based on self-reports of vision and hearing abilities as well as experienced disability since no other measurements of sensory functions were available. This method of collecting data provides information about perceived difficulties in everyday life and is clinically relevant for analysing the association between sensory functions and disability. Although self-reported hearing and vision assessments can serve as useful indicators of an individual’s sensory status, they may not accurately identify all cases of sensory impairment [50–52]. However, these questions have been validated in previous research for their suitability in identifying sensory loss among older adults [50, 52, 53]. In contexts where direct assessments like pure-tone audiometry and visual acuity tests are not feasible, self-reported data becomes a viable alternative. It is also important to consider that relying solely on self-reported measures might not effectively detect older adults experiencing early stages of sensory decline. However, one study [54] found that the self-report of hearing difficulties has been shown to be associated with all major domains of hearing aid outcomes, including help-seeking, uptake of amplification, use of hearing aids in daily life, and satisfaction with care. Furthermore, another study [55] suggested that self-reported vision evaluations may be more appropriate for determining the risk of cognitive impairment in daily life as opposed to visual acuity measures. In our study, the self-reported assessment of sensory abilities, raises an important consideration regarding the use of hearing aids and glasses by participants. It is essential to recognize that the presence of such corrective measures can significantly influence the participants self-reported sensory status which presents a potential challenge in accurately capturing the true extent of sensory difficulties among older adults. For instance, individuals who rate their hearing or vision as “good” while using hearing aids or glasses may still experience substantial impairment without these devices. Nevertheless, we acknowledge that our study may not fully capture the nuanced interplay between sensory impairments and the mitigating effects of assistive devices. As such, while our findings shed valuable light on the prevalence and associations of sensory difficulties in older adults, they should be interpreted in light of this limitation, recognizing the potential for underestimation of the true sensory challenges faced by this population. Further research exploring the interrelationship between assistive devices, self-reported sensory status, and objective sensory assessments is needed to enhance our understanding of sensory health in older age.

Our findings are consistent with previous studies that have shown a higher prevalence of vision loss among women and hearing loss among men, particularly in older age groups, despite different prevalence estimates and methodologies. In Europe, the prevalence of self-reported vision difficulties has been found to be higher in women and older people [56], while in the US the prevalence of self-reported hearing difficulties was found to be higher in men and older adults [57], which is consistent with our findings. Similarly, in the US, some studies using objective measures [14, 45] have found that vision loss is more common in women aged 60 and above, while hearing loss is more prevalent in men [58, 59]. Regarding social inequalities measured by level of education, the present study shows that education is inversely associated with sensory limitations and was consistent with results from previous studies that employed objectives measures of impairment such as Agrawal (2008), which found that hearing loss was most common in adults aged 70 and older, as well as in those with lower levels of education and ethnic minorities [32]. The variation in sensory difficulties between Swedish and foreign-born individuals, with hearing issues more prevalent among the latter, may suggests several potential factors at play. One explanation could be lower usage of hearing aids among foreign-born individuals, possibly due to disparities in healthcare access, health literacy [60], and cultural factors. Additionally, differences in occupational exposures [61] and linguistic barriers may contribute to these disparities. However, it’s essential to interpret these findings cautiously due to the limited sample size of foreign-born individuals and the subsequent uncertainties around the estimates, as shown by the wide confidence intervals. Further research with larger, more diverse samples is needed to explore these factors comprehensively.

According to the WHO, the prevalence of hearing loss in Europe ranges from 11 to 28%; however, in people aged 65 and above, it can range from 28 to 78% across different European countries [15]. In the present study, the prevalence of self-reported dual sensory difficulties in people aged 70 and above was 6.1%, in addition to 18.9% for hearing and 10.3% for vision difficulties. The implications of an aging population are profound, both within Sweden and on a global scale. As individuals age, the prevalence of sensory difficulties, including hearing and vision impairments, increases as identified in this study and previous ones [62–64]. This trend is not only a public health concern but also has wider social and economic implications. In Sweden, as in many other countries, the older segments of the population are growing, which underscores the importance of understanding and addressing age-related sensory difficulties. By exploring this aspect, we can provide insights into the potential healthcare needs and support systems required for older adults.

Several theories have been proposed to explain the observed sex differences in age-related sensory losses. It has been suggested that men are more likely to be exposed to loud noise in their occupational or leisure activities, which can lead to noise-induced hearing loss [65]. Additionally, men may be more likely to engage in unhealthy behaviours, such as smoking, which has been shown to be associated with hearing loss [66]. On the other hand, hormonal differences between sexes may influence the development of vision loss. Oestrogen has been indicated to have protective effects on vision [67] and hearing [68], while a decline of oestrogen levels during menopause may increase the risk of developing age-related eye disease [67] and hearing loss [69].

Education may also play a role in the development of age-related sensory loss as it may lead to increased access to healthcare and preventive measures, such as hearing aids, glasses and eye surgeries, which can help detect and prevent sensory losses. Moreover, it is possible that higher levels of education may be linked to differences in the exposure to loud noises in the work environment, as individuals with higher education may have more specialised occupations. Education can also lead to healthier lifestyle and behaviours that can help preserve sensory functions and may also lead to cognitive reserve and greater cognitive ability, which can help offset the effects of age-related sensory losses [70]. These theories have not been tested in this study; however, future research should explore the mechanisms underlying sensory losses and the factors associated with it.

Sensory losses are a significant global health issue that have a major impact on quality of life and can lead to social depression and reduced productivity. They are also important causes of years lived with disability (YLDs) and a significant burden on healthcare systems. Hearing loss is responsible for over 40 million YLDs globally and is ranked as the third most common cause of YLDs in the Global Burden of Disease [18] study. However, when explicitly modelled for people older than 70 years, age-related and other hearing loss was the leading cause of YLDs globally [18]. Although the age-adjusted prevalence of blindness was reduced by 27% between 1990 and 2020, the prevalence of moderate and severe vision loss increased slightly [71]. As the world population grows and ages over the coming decades, the need for hearing and vision care will rise. Sensory losses can often be prevented or modified (e.g., treatment of correctable visual loss, use of vision and hearing aids, cochlear implants) and targeting sensory function interventions in aging populations could potentially prevent of delay cognitive decline.

## Conclusion

In this cross-sectional study, the prevalence of self-reported sensory difficulties differs by migration status, sex, and education in Sweden in 2019/2020. Moreover, the prevalence of hearing, vision and dual-sensory loss increased with age. Sensory losses have implications for the increased risk of an early onset of disability, mental health problems and increased burden on the health care system. Due to the numerous risks associated with these losses and the possible consequences of impairment for affected people, their dependents, and society, interventions to help older adults prevent or postpone the onset of vision or hearing loss are needed. More research is needed to understand the causal mechanisms by which social inequalities are related to sensory losses in the older population and provide information useful for policy formulation aimed at risk reduction.

## Data Availability

The SHARE data can be accessed free of charge here: https://share-eric.eu/data/, however the datasets used in the study cannot be provided due to the rules set by the Swedish Ethical Review Authority.
