# Correction: A descriptive epidemiological study of the prevalence of self-reported sensory difficulties by age group, sex, education, disability, and migration status in Sweden in 2020

**DOI:** 10.1186/s12889-024-21186-1

**Published:** 2025-01-07

**Authors:** Andreea-Corina Badache, Elina Mäki-Torkko, Stephen Widen, Stefan Fors

**Affiliations:** 1 School of Health Sciences, Örebro University, Fakultetsgatan 1, Örebro, 701 82 Sweden; 2 Swedish Institute for Disability Research, Örebro, Sweden; 3 School of Medical Sciences, Örebro University, Örebro, Sweden; 4 Audiological Research Center, Faculty of Medicine, and Health, Örebro University, Örebro, Sweden; 5 Aging Research Center, Karolinska Institutet and Stockholm University, Stockholm, Sweden; 6 Centre for Epidemiology and Community Medicine, Region Stockholm, Stockholm, Sweden; 7 Department of Public Health Sciences, Stockholm University, Stockholm, Sweden

**Correction** **:** **BMC Public Health 24, 2773 (2024)**

**https://doi.org/10.1186/s12889-024-20217-1**

The original publication of this article [1] contained an incorrect "Ethics approval and consent to participate" section. The incorrect and correct information is listed in this correction article. The original article has been updated.

Incorrect

To analyze SHARE data, the study has received an ethical approval from the Swedish Ethical Review Authority, Dnr 2019–06445 which has been updated Dnr 2023-04400-02.

Correct

To analyze SHARE data, the study has received an ethical approval from the Swedish Ethical Review Authority, Dnr 2019-06445.
